# Maqui (*Aristotelia chilensis (Mol.) Stuntz*): A Natural Antioxidant to Improve Quality of Meat Patties

**DOI:** 10.3390/antiox11071405

**Published:** 2022-07-20

**Authors:** Lidiana Velázquez, John Quiñones, Karla Inostroza, Gastón Sepúlveda, Rommy Díaz, Erick Scheuermann, Rubén Domínguez, José M. Lorenzo, Carla Velásquez, Néstor Sepúlveda

**Affiliations:** 1 Centro de Tecnología e Innovación de la Carne (CTI-Carne), Facultad de Ciencias Agropecuarias y Forestales, Universidad de La Frontera, Temuco 4780000, Chile; l.velazquez01@ufromail.cl (L.V.); john.quinones@ufrontera.cl (J.Q.); g.sepulveda10@ufromail.cl (G.S.); rommy.diaz@ufrontera.cl (R.D.); c.velasquez12@ufromail.cl (C.V.); 2 Programa de Doctorado en Ciencias Agroalimentarias y Medioambiente, Universidad de La Frontera, Temuco 4780000, Chile; 3 Departamento de Ciencias Agropecuarias y Acuícolas, Facultad de Recursos Naturales, Universidad Católica de Temuco, Temuco 4780000, Chile; kinostroza@uct.cl; 4 Departamento de Ingeniería Química, Facultad de Ingeniería y Ciencias, Universidad de La Frontera, Temuco 4780000, Chile; ericks@ufrontera.cl; 5 Centro Tecnológico de la Carne de Galicia, Rúa Galicia No. 4, Parque Tecnológico de Galicia, San Cibrao das Viñas, 32900 Ourense, Spain; rubendominguez@ceteca.net; 6 Área de Tecnoloxía dos Alimentos, Facultade de Ciencias de Ourense, Universidade de Vigo, 32004 Ourense, Spain

**Keywords:** natural additives, functional food, beef patties shelf-life, lipid oxidation, oxidative stability, meat products, healthy meat

## Abstract

*Aristotelia chilensis* is an endemic shrub of the South Pacific with high concentrations of bioactive compounds in its leaves and, therefore, it is highly valued. The effect of *Aristotelia chilensis* leaf powders (maqui leaf powders; Ma) on the quality and shelf life of beef patties during 7 days of storage was investigated. Five beef patties treatments were prepared: (1) Control without antioxidants (CT); (2) Beef patties with synthetic antioxidants plus color (250 mg/kg) (PL); (3) Beef patties with 500 ppm of maqui leaf powders (Ma500); (4) Beef patties with 1000 ppm of maqui leaf powders (Ma1000); and (5) Beef patties with 2000 ppm of maqui leaf powders (Ma2000). The quality of the beef patties was evaluated on day 0 and day 7 of storage by physicochemical analysis (moisture, ash and lipid content, color, pH, fatty acid profile and lipid oxidation) and organoleptic analysis. The addition of maqui leaf powders did not produce changes in the proximate composition of the beef patties. The pH for all treatments showed a range of 5.50–5.75 and significant differences (*p* < 0.05) were observed at the beginning and end of storage. The pH of the control beef patties increased during storage while the pH of the beef patties with synthetic and natural antioxidants decreased. Redness (a*) was the color indicator that was mostly affected by the inclusion of 1000 ppm and 2000 ppm powders. High lipid oxidation was observed in control samples on the seventh day of storage due to the high percentage of fat used in the formulation and the absence of any antioxidant. However, the Ma500, Ma1000, and Ma2000 treatments presented the lowest lipid oxidation rates (42.05%, 40.29%, and 43.14%, respectively) in comparison with the synthetic antioxidant (52.23%). This lipid inhibition is related to the strong antioxidant activity (29.75 µg/mL IC_50_ DPPH) of the maqui leaf powder due to its high content of total polyphenols (148.76 mg GAE/g), mainly characterized by having great amounts of hydroxybenzoic acids (82.5 mg GAE/g), flavonoids (7.1 mg QE/g), and hydroxycinnamic acids (3.7 mg CAE/g). Although minimal variations were observed in some individual fatty acids, and despite the trend to decrease MUFA and increase SFA with the maqui leaf powder addition, these differences were minimal and, according to the nutritional indices results, without any influence on the nutritional quality of the beef patties. The organoleptic analysis showed that the addition of maqui leaf powders did not affect the general acceptability of the new formulations. This study reports for the first time the substitution of synthetic antioxidants with *Aristotelia chilensis* leaves extract. Based on the results, it can be concluded that this ingredient can be used as an alternative for the production of raw meat products with clean labels.

## 1. Introduction

For several years now, the meat industry has assumed the role of providing consumers with healthier products, in accordance with their new needs aimed at taking care of their health based on a good diet [1]. This challenge is even greater when agro-industrial production must be conducted with a sustainable approach. To this end, some strategies have been studied and implemented, such as improving meat products’ lipid profile and nutritional indices by replacing animal fats with vegetable oils [2,3,4,5,6,7,8]. However, improving the lipid profile of meat products generates other technological drawbacks, as increasing the amount of monounsaturated fatty acids (MUFA) and polyunsaturated fatty acids (PUFA) increases oxidative instability and decreases the shelf life of the products, due to the propensity of these fatty acids to oxidize more rapidly [9,10]. For this reason, several authors conclude that this strategy should be accompanied by the incorporation of antioxidants that prevent accelerated deterioration of these new products [11,12,13,14]. While both synthetic and natural antioxidants can be used to improve the oxidative stability of meat products [15,16], natural antioxidants are preferred by informed consumers and have higher consumer acceptance [17,18]. Studies have evaluated the use of different plant extracts for improving the storage quality of meat products, for example, Bellucci et al. [19] evaluated the antioxidant action of *Hylocereus costaricensis* extracts in beef patties with the replacement of animal fat by *Cyperus esculentus* oil, which significantly reduced lipid oxidation in these products. Similarly, the use of pitanga (*Eugenia uniflora* L.) leaf extracts [13] or guarana (*Paullinia cupana*) seeds extracts [20] in pork beef patties also presented promising results. In these studies, the authors observe a clear and strong antioxidant capacity of the extracts and an extension of beef patties’ shelf life when they were added. Moreover, in a more recent study, the same research group evaluated both extracts in lamb beef patties with fat replacement by chia oil hydrogel emulsion, and concluded that the use of 250 ppm of each extract is effective against color deterioration and lipid and protein oxidation, without impairing the sensorial characteristics, representing a promising alternative to replacing synthetic antioxidants by natural products in lamb beef patties [21]. The replacement of synthetic antioxidants linked to health damage by plant polyphenols with antioxidant, antimicrobial, and therapeutic potentials has been widely studied [11,13,22]. To this end, the impact of several plant extracts on the storage quality of meat products has been studied [21,22,23], however, the effect of Maqui on the storage stability of meat products has not been reported and requires scientific attention. Additionally, the use of natural antioxidants from endemic leaves in the reformulation of meat products, such the case of the present study, was extensively discussed in a recent review [24].

Maqui (*Aristotelia chilensis (Mol.) Stuntz*) is an evergreen shrub native to the South Pacific and highly valued worldwide, due to its high concentrations of bioactive compounds. In Chile, the annual production of maqui fruit is estimated at 130 thousand tons, mainly in dried form, and more than 80% of the production is exported to countries such as South Korea [25]. Maqui fruits are commonly consumed as snacks and used for the artisanal manufacture of jams and liqueurs. At the industrial level, they are used for the production of natural colorants, ice cream, jams, juices, and in the pharmaceutical industry—for the production of antioxidant supplements due to the high concentrations of anthocyanins present in the fruit [26]. There is ample evidence indicating the antioxidant value of maqui leaves compared to other parts of the plant. Previous studies have compared the antioxidant activity and polyphenolic concentration of maqui fruits, stems, and leaves, concluding that the leaves stand out in their polyphenolic concentration of 78.5 ± 0.43 mM EAG and antioxidant power of 189.50 ± 10.25 mM EAG [27]. This is attributed to its bioactive compounds such as alkaloids, flavonols, phenolic acids, and tannins [28]. However, scientific evidence of the antioxidant capacities of maqui leaves in food matrices, particularly in meat products, is scarce. With this in mind, and taking into account the need to explore new natural sources of compounds and natural additives that increase the shelf life of meat products, the trial of this extract is proposed as a promising alternative for the meat industry. In addition, it is also worth highlighting the enormous opportunity that represents the possibility of using an agro-food by-product as a potential source of natural additives, as has been widely evaluated in other types of by-products [12,29].

Therefore, this study aimed to evaluate the effect of maqui leaf powders as a natural antioxidant on the quality and stability of beef patties. This was observed through the impact that this extract had on the chemical composition, physicochemical parameters, oxidative, and sensory attributes of beef patties, as well as its possible use as a shelf-life extender.

## 2. Materials and Methods

### 2.1. Plant Material

Maqui (*Aristotelia chilensis (Mol.) Stuntz*) leaves sampled in May 2021 at the Maquehue Experimental Field of the Universidad de La Frontera, Chile (38°50′16.01″ S, 72°41′39.98″ W) were used. The samples were washed with distilled water and dried in an oven at 35 °C (BINDER ED25. Germany) until they reached constant weight. Then, they were ground in an ultracentrifugal mill (Retsch model ZM 200. Germany) passing them through an 80 µm sieve. The maqui leaves powders (MLP) were stored in polyethylene bags at −21 °C until further use.

### 2.2. Maqui Leaf Powder Characterization: Total Phenolic Content, Main Phenolic Compounds and Antioxidant Activity

The determination of the total polyphenol content was performed following the methodology described by Singleton [30] with some modifications. The principle of this method is based on the reaction at basic pH between phenolic compounds and the Folin–Ciocalteu reagent, giving rise to a blue compound that absorbs at 760 nm. First, 40 µL of extracts was taken into a 4 mL vial containing 3160 µL of distilled water. Next, 200 µL of Folin–Ciocalteu reagent (2N) was added and the mixture was completely vortexed. Then, 600 µL of Na_2_CO_3_ (20%) was added. The mixture was incubated in the dark at 25 °C for 2 h. The absorbance at 760 nm was measured. The results were expressed as mg GAE/g dry sample.

In addition, the main groups of phenolic compounds present were also analyzed by HPLC equipped with a DAD detector. Following extraction of the compounds using methanol:water (50/50), the compounds were analyzed using a Nucleodur 100-C18 column (5 µm, 4.6 × 250 mm). The mobile phases were Phase A: acetonitrile, Phase B: water and Phase C: formic acid. The flow of the mobile phase was constant at 0.8 mL/min and the gradient used in the analysis varied between the beginning of the analysis (Phase A (3%), Phase B (87%) and Phase C (10%)) until increase phase A to 50% (Phase A (50%), Phase B (40%) and Phase C (10%)) at the end of the analysis. The total analysis time was 50 min, keeping the column thermostated at 40 °C throughout the process. Hydroxybenzoic acids derivatives were determined at 275 nm (standard: gallic acid; quantified as mg gallic acid equivalents/g), hydroxycinnamic acid derivatives at 320 nm (standard: caffeoylquinic acid; quantified as mg caffeoylquinic acid equivalents/g) and flavonoids at 360 nm (standard: quercetin; quantified as mg quercetin equivalents/g).

The antioxidant activity of maqui leaf powder was evaluated according to the α,-Diphenyl-ß-picrylhydrazyl (DPPH^•^) [31]. This method determines the antioxidant capacity according to the free radical scavenging capacity of the antioxidant compounds present in the extracts. To this end, 100 µL of standard or extract was mixed with 3.9 mL of DPPH solution (5.6 × 10^−5^ M) (Sigma-Aldrich). It was incubated at 37 °C for 10 min in the dark and the *absorbance* was read at 515 nm. The results were expressed as a percentage of free radical inhibition (*%FRI*). Trolox was used as a reference antioxidant standard (0–200 µg/mL) and the antioxidant activity of maqui leaf powder was compared with that of the standard based on IC_50_ (µg/mL).

(1) $$\%\mathrm{FRI}=\frac{\text{Absorbance}{\;}_{t=0\min}-\text{Absorbance}{\;}_{t=30\min}\;}{\text{Absorbance}{\;}_{t=0\min}\;}\times 100$$

Three different concentrations of maqui extracts (325, 155, and 10 µg/mL) were tested. Measurements were performed in triplicate using a UV Vis Genesis 10 S.

### 2.3. Preparation of the Beef Patties

Beef primal cuts were purchased at the local market on the same day of the trial. The bacon and synthetic antioxidant were provided by an artisanal jerky production plant in the Araucanía Region. Five batches of beef patties were prepared: (a) Control without antioxidants; (b) With 250 mg/kg commercial antioxidants Plus color^®^, (c) 500 mg/kg MLP, (d) 1000 mg/kg MLP, and (e) 2000 mg/kg MLP. The dough for the beef patties was made by mixing beef meat (70%) and bacon (30%), using an 8 mm disc. Then ice (5%), salt (1.5%), and the rest of the previously ground seasonings were added and mixed for 5 min. The dough was divided into 100 g portions and placed in a beef patties mold (10 cm diameter and 1 cm high). A total of 26 beef patties per treatment were produced and portioned for shelf-life analysis. On day 0, 8 beef patties per treatment were taken for proximate composition analysis and 10 beef patties for sensory analysis. The remaining beef patties were vacuum-packed in polyethylene bags and stored at 4 °C for 7 days. This experiment, with the same ingredients and processing steps, was replicated twice.

### 2.4. Proximate Composition

Moisture and ash contents were determined according to the procedures recommended by ISO procedures [32,33], while the determination of total lipids was performed according to the Folch technique [34].

### 2.5. Determination of pH and Color of the Beef Patties

The pH of the beef patties (6 replicates per treatment, manufacture replicate, and sampling day) was measured using a digital pH meter (Hanna Instruments Inc., Woonsocket, RI, USA) previously calibrated, making punctures at 6 different points of each beef patties. The color of the patties was measured on day 0 and day 7 with a portable colorimeter CR-10 (Konica Minolta Sensing, Inc., Tokyo, Japan) with the CIELab system, equipped with a pulsed xenon arc lamp filtered at D65 illumination conditions, 0° viewing angle geometry, and 8 mm aperture size.

### 2.6. Fatty Acid Profile

For fatty acid analysis, the beef patties were minced with a domestic mincer to homogenize the samples. Lipids were extracted according to the methodology proposed by Folch et al. [34]. The meat patty samples were homogenized by stirring in a solution of n-hexane:isopropanol (2:1 *v/v*) (Merck, Darmstadt, Germany). The mixture was centrifuged at 3000 rpm for 10 min. The supernatant was transferred, and the solvent was evaporated at constant temperature and pressure at a rotary evaporator. The total lipid sample was then dried with nitrogen gas and the weight difference was determined to obtain the total fat content. Fatty acid methylation (fatty acid methyl esters; FAME) was carried out using 800 µL of n-hexane (Merck, Darmstadt, Germany) and 1.3 mL of 2 N potassium hydroxide in methanol (Merck, Darmstadt, Germany), added to each sample, and then magnetically stirred for 30 min. The supernatant was filtered with 0.5 g anhydrous sodium sulfate (Merck, Darmstadt, Germany) and centrifuged at 2000× *g* at room temperature for 5 min. FAMEs were analyzed using a gas chromatograph (Clarus 500, Perkin Elmer, MA, USA) coupled with a flame ionization detector (FID), split injection mode, and automatic sampler. FAME separation was performed with an SPTM 2380 fused silica capillary column (60 m × 0.25 mm × 0.2 µm film thickness) (Supelco, Bellefonte, PA, USA) by injecting one microliter of FAME extract. A gradient program was used for column temperature: the initial temperature was set at 150 °C, after 1 min, the temperature was increased at a rate of 1 °C min^−1^ to 168 °C, held for 11 min, then increased at 6 °C min^−1^ to 230 °C, and this temperature was held for 8 min. The detector and injection port temperature were 250 °C, and nitrogen was used as the carrier gas. Individual FAMEs were identified by retention time using a standard 37-component FAME Mix C4-C24 (Supelco, Bellefonte, PA, USA). For the identification of conjugated linoleic acid (CLA) isomers, standard octadecadienoic acid, and conjugated methyl ester (CLA Sigma-Aldrich, Milwaukee, WI, USA) were both analyzed under the same chromatographic conditions.

### 2.7. Lipid Oxidation (TBARs)

The determination of thiobarbituric acid reactive substances (TBARs) was performed according to Vyncke procedure [35] with some modifications. The degree of oxidation of fats in this method is measured through a chromogenic compound formed as a result of the reaction between malonaldehyde (MDA) and thiobarbituric acid in an acid medium. Malonaldehyde is the main compound formed as a result of the oxidation of lipids, especially unsaturated lipids. Briefly, 5 g of sample was weighed and mixed for 10 min with 15 mL of 7.5% trichloroacetic acid (TCA) containing 0.1% propyl gallate and EDTA. It was filtered and to 5 mL of that filtrate, 5 mL of 0.02 M 2-thiobarbituric acid was added. It was incubated for 40 min in a boiling water bath. It was cooled and the absorbance was measured at 538 nm. TBARS rates were calculated using a standard curve of 1,1,3,3,3 trihydroxypropane (TEP) (Sigma-Aldrich) and the results expressed as mg MDA/kg of sample.

### 2.8. Sensory Analysis

The sensory evaluation of the patties on day 0 was carried out by a panel formed by a trained panel consisting of 8 evaluators. The sensory analyses were performed under controlled conditions in a room with white light in the Meat Center for Technology and Innovation of the University of La Frontera, according to NCh-ISO6658:2016 [36]. Each beef patties wrapped in aluminum foil was cooked in an electrical contact grill large double S + S/S + S (MilanToast, Sulbiate, MB, Italy) pre-heated at 150 °C and cooked the beef patties until reaching a central temperature of 70 °C determined by a handled probe. Samples were cut into cubes (1.5 cm × 1.5 cm × 1.5 cm), wrapped in aluminum foil, and assigned a code number of three digits to identify each sample. Then, they were immediately presented to the assessors in plastic dishes. Unsalted crackers and mineral water were provided to the consumers to clean their mouths between samples. A 10 semi-structured points scale was used (0 = low, 10 = high) [37] to score the degree of the following attributes: tenderness, juiciness, odor, flavor, and overall acceptability. All the assessors were from the Meat Center for Technology and Innovation of the University of La Frontera with experience in evaluating animal product quality.

### 2.9. Statistical Analysis

Statistical analysis was performed with IBM SPSS Statistics 23 (IBM Corporation, Somerset, Armonk, NY, USA). The results were expressed as the mean ± standard deviation. The normality of the variables was confirmed by the Shapiro–Wilk test and the homogeneity of variances by Levene’s test. Analysis of Variance (ANOVA) test was performed where the 4 treatments were compared on days 0 and 7 of analysis. When significant differences were detected between groups, Tukey’s test was performed. The significance level was set at (*p* < 0.05). To determine the effect of the treatments on the organoleptic characteristics, a PCA analysis was performed using the Factoshiny package [38] by means of the R.4.0.5 software.

## 3. Results and Discussion

### 3.1. Total Polyphenol Content and Antioxidant Activity of Maqui Leaf Powder

The results of the content of total polyphenols, main phenolic compound families, and antioxidant capacity of the maqui leaf powders are shown in Table 1. Maqui leaves showed a high concentration of polyphenols 148.76 mg GAE/g dm. These results are superior to those reported by Rubilar et al. [39] who quantified 69 mg GAE/g dm in ethanolic extracts of *A. chilensis* leaves. In our study, maqui leaf powder polyphenols were characterized by high amounts of hydroxybenzoic acids (82.5 mg GAE/g) and less content of flavonoids (7.1 mg QE/g) and hydroxycinnamic acids (3.7 mg CAE/g). This agrees with those reported by other authors who specified that the main phenolic groups in plants are phenolic acids (both, hydroxybenzoic and hydroxycinnamic acids) and flavonoids [40]. As is known, the polyphenolic concentration can vary widely from one plant to another, influenced by extrinsic and intrinsic factors of the plants. Regarding the work of Rubilar et al. [39], there was variability in the place and time of year in which the sampling was carried out. In addition, in this research, we used an S/L ratio (1:10) and the ultrasound extraction method that favors the extraction kinetics, increasing the yield of soluble solids and therefore the concentration of total polyphenols [41,42].

Regarding antioxidant activity, ethanolic extracts from *A. chilensis* leaves showed strong antioxidant activity against DPPH. In addition, higher antioxidant activity was observed as the concentration of polyphenols increased. The inhibition percentage increase was from 15% with 10 µg/mL, to 94% with 325 µg/mL. Moreover, 29.75 ± 0.06 µg/mL of *A. chilensis* was needed to inhibit 50% of the DPPH radical. Munoz et al. [43] reported an antioxidant activity in *A. chilensis* leaves for aqueous and methanolic extracts of 12.1 and 9.7 µg/mL, respectively. Additionally, our results are very similar to those obtained by Rubilar et al. [39] that hydroethanolic extracts from *A. chilensis* leaves had an antioxidant activity of 29.4 µg/mL. In this study, the authors also reported an IC_50_ of maqui leaf crude extract of 8 µg/mL [39]. These data confirm, on the one hand, that the solvent used exerts an enormous influence on the extraction of antioxidant compounds, and therefore, on the antioxidant capacity of the extract, and on the other hand, that the maqui leaf powder has a good antioxidant capacity and a high content of total phenolic compounds.

### 3.2. Proximate Composition

Table 2 summarizes the proximate composition of the beef patties. The inclusion of maqui leaf powder had no significant effect (*p* > 0.05) on moisture (61.10–63.22%), fat (21.50–22.50%) and ash (1.78–2.30%) contents. These results agree with those of other authors who evaluated the effect of pitanga leaf extracts [13,21] or mesquite leaf powder [44] on pork beef patties and conclude that the reformulation had no effects on beef patties chemical composition. However, the values obtained for moisture, fat, and ash by all these studies vary between them, and as expected, these changes are related to the initial formulation variations (mainly due to the different proportions of meat and fat used in the beef patties manufacture).

In contrast, the reformulation of pork beef patties with walnut leaf powders produces a significant increase in the moisture retention in the product [45]. Similarly, the addition of radish leaves and roots to pork beef patties formulations also produced a significant increase in the moisture and ash contents [46]. These increases in the moisture content could be related to the fiber present in these leaf powders, which have favored water retention. Moreover, the dehydrated leaf powders could also contribute with a high mineral amount to the total ash content of beef patties, and explain the results obtained [46]. Another study reported a significant increase of ash in beef patties reformulated with moringa leaf powder, due to the previous explanation, but they also found a significant decrease in moisture content [47]. They attributed the decrease in moisture amount to the increase in the total soluble solids.

### 3.3. pH and Color

In general, the pH of our reformulated beef patties showed a range of 5.50–5.75 (Table 3). These values below 6 indicate a normal range for beef patties [48]. From day 0, significant differences were observed between the control treatments (5.50) and the treatments with synthetic (5.76) and natural (5.62–5.75) antioxidants. The control beef patties showed a significantly lower pH (*p* ≤ 0.05) than the rest of the treatments. Similarly, other studies also found that control beef patties presented the lowest pH values after production (day 0) in comparison with those reformulated using leaf powders [13,21,45], while in other cases, no differences were found [46]. During storage, there was a general tendency for the pH of the samples with synthetic and natural antioxidants to decrease. These results agree with those reported by previous studies [13,21], which observed that pH in beef patties with pitanga leaf extracts decreased significantly on the seventh day of storage. Additionally, it has been reported that during 12 days of storage of chicken beef patties, moringa leaf powders (100 g/kg) favored pH decrease from 5.9 to 5.6, approximately [48], and pork beef patties reformulated with radish leaves and root powder also presented lower values of pH (<5.3) after 14 days in comparison with the values of day 0 (>5.5) [47]. Commonly, pH decreases during storage at 4 °C are associated with inhibition of pathogenic bacteria development and formation of biogenic amines [46]. In other words, some compounds present in acidic (ionized) media are able to more easily penetrate the cytoplasmic membrane of bacteria or inactivate enzymatic activity within the cell. In addition, bacteria growing in acidic media compete for nutrients, oxygen, and adhesion sites preventing the development of pathogenic bacteria [49]. Conversely, the pH of the control samples, which at the beginning of storage was lower with respect to the rest of the treatments, increased considerably on the seventh day (Table 3). In meats and meat products, this fact is an indicator of microbial growth that favors amino acid degradation and the release of ammonia (alkaline pH) to the medium [50]. Unfortunately, microbiological tests were not conducted in this study. Therefore, in future research, microbiological analyses should be conducted to test the antimicrobial effect of maqui leaf powders on beef patties.

On the other hand, oxidative reactions are the main cause of the loss of characteristic color of meat products, especially related to chemical and structural changes of the myoglobin molecule [9,51,52]. In this work, changes in color were observed by colorimetry using the CIELab system. This method delivers several parameters that are linked to myoglobin. For example, redness (a*) is an indicator of the concentration of the myoglobin molecule. In addition, yellowness (b*) allows us to estimate the chemical state of myoglobin, and lightness (L*) is related to the state, size, and position of the muscle fibers that condition the degree of reflection and absorption of the light spectrum, together with the presence of free and bound water [53,54]. As expected, our results indicate that the addition of 1000 ppm and 2000 ppm of maqui leaf powder affected the redness (a*) of the beef patties from the beginning of storage, showing significantly lower values (19.15 and 18.45), with respect to the control, plus color and Ma500 treatments (23.70, 23.55 and 22.90, respectively) (Table 3). However, they still presented acceptable values, since it is estimated that when redness reaches values between 4.6–10.8, the product is perceived as brown [55]. These results agree with those of Zamuz et al. [48]. In this case, the authors reported that the color on the surface of beef patties was modified by the inclusion of extracts at 1000 ppm of strawberry leaves. On the other hand, in contrast to the redness, the yellowness (b*) and lightness (L*) of our beef patties was not affected by the inclusion of maqui leaf powders at the beginning of storage. However, at 7 days, redness, yellowness, and lightness had significantly decreased in all treatments, although it was more marked in treatments with maqui powder. Similarly to our results, several authors reported a significant reduction in the redness of beef patties during storage [44,45,48] both, in control and samples with leaf powders or leaf extracts, and also was found a decrease in yellowness [45,48] and luminosity [45,48] in samples with leaf extracts/powders during the first 7–15 days. In the present research, the effects of maqui powder on color instability in meat can be attributed to chlorophyll and other antioxidant compounds present in the leaves of the plants [45,55]. In other words, the green color of the leaves may be a source of color variation, as it is completely different from the natural color of the meat. In addition, the oxidative processes inherent to meat and meat products can favor color loss. For example, protein oxidation has also been reported to have an effect on coloration, as it leads to a loss of surface water holding capacity and as a result increased light scattering [20,52]. In the treatments with synthetic antioxidants, the redness was preserved 35.37% versus 33.15% of the control beef patties. In treatments with Ma500, Ma1000, and Ma2000 redness was retained 29.61%, 31.96%, and 25.85%, respectively. Several factors influence the discoloration of meat and meat products during storage (temperature, lighting, relative humidity, microbial load, and lipid or protein oxidation) and all of them have a direct effect on myoglobin, therefore, strategies to preserve meat color should involve delaying pigment oxidation and/or enhancing the reduction of oxidized pigment [56].

### 3.4. Lipid Oxidation

**Figure 1:**
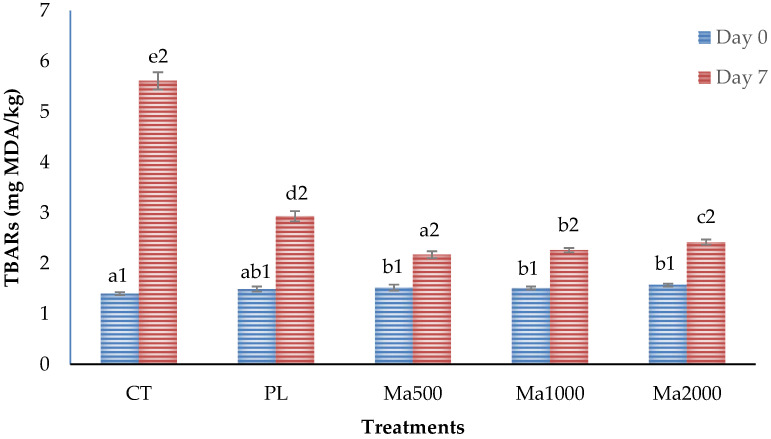
Effect of antioxidant treatment and storage time on the lipid oxidation (TBARs value) of beef patties. CT: control beef patties formulated without antioxidants; PL: beef patties formulated with synthetic antioxidant Plus color^®^; Ma500: beef patties formulated with 500 mg/kg of maqui leaf powder; Ma1000: beef patties formulated with 1000 mg/kg of maqui leaf powder; Ma2000: beef patties formulated with 2000 mg/kg of maqui leaf powder. (^a–e^) Different letters in the same storage time indicate significant differences between treatments (*p* < 0.05; Tukey’s test); (^1,2^) Different numbers indicate, within the same treatment, differences between storage time (*p* < 0.05; ANOVA).

Lipid oxidation in meat products during storage leads to loss of nutritional quality, color, texture, flavor, and aroma, and is the main cause of non-microbial spoilage in meats and meat products [9,57]. Most studies of lipid oxidation in meats are performed using the thiobarbituric acid reaction technique (TBARs) quantifying the secondary products of oxidation (malonaldehyde) as these are mainly responsible for undesirable odors and flavors [58]. The results of this research showed significant differences between the treatments for the combined effect of antioxidants and storage time (Figure 1). Lipid oxidation of all samples increased with storage, but beef patties without antioxidants reached much higher malonaldehyde concentrations (5.61 mg MDA/kg) than the other groups (between 2.17 and 2.93 mg MDA/kg). The same trend was observed by other authors in beef patties, where control beef patties presented a dramatic increase of TBARs, while this increase was less pronounced in the reformulated samples (with natural or synthetic antioxidants) [13,20,45,46,47,58]. A significant increase in TBARs values with the storage time is expected since the action of endogenous and microbial prooxidant enzymes and the release of heme iron from the myoglobin (which catalyze autoxidation) is favored [9]. Additionally, it is important to highlight that the beef patties with maqui leaf powders (Ma500, Ma1000, and Ma2000) presented much lower percentages of MDA (42.05%, 40.29%, and 43.14%, respectively) than those formulated with the synthetic antioxidant Plus color (52.23%). In other words, maqui leaf powders were able to significantly delay lipid oxidation, with the treatment with 1000 ppm powder showing the best results. These results correlate positively with the antioxidant capacity shown by maqui extracts in the “in vitro” studies discussed in Section 3.1 of this manuscript. Furthermore, we confirmed that the antioxidant capacity was proportional to the level of powders used and higher than that of the synthetic antioxidant Plus color. This effect can be attributed mainly to hydroxybenzoic acid, which is the main antioxidant found in the leaves used in this research. In this sense, the important role of hydroxybenzoic acid as an antioxidant in foods has been described [59,60]. The mechanism of its antiradical action is centered on its ability to release hydrogen atoms. Normally, the resulting intermediates can interact with each other and/or with free radicals inside reactions, which contributes to the observed antioxidant effect [59,60].

It is not possible to compare our results with other studies using maqui extracts. In fact, this research provides a first approach to the use of maqui leaves to extend the shelf life of meat products. However, the antioxidant capacity in burgers reformulated with other vegetal species such as leaf lotus, walnut leaf powder, and chestnut leaf has been confirmed to be equal or superior to that of synthetic antioxidants such as BHT [45,48,58]. Thus, our results are in perfect agreement with those obtained by other authors and with the scientific evidence.

### 3.5. Fatty Acid Profile

Analysis of the fatty acid (FA) profile at the beginning and end of storage showed that beef patties were mainly composed of MUFA (46.10–48.35 g/100 g fatty acids) followed by 38.55–42.36 g/100 g FA of saturated fatty acids (SFA) and 11.55–13.30 g/100 g FA of PUFA. The predominant fatty acid in all treatments was oleic acid (C18:1 n − 9) (42.37–44.24 g/100 g FA), followed by palmitic acid (C16:0) (21.95–23.87 g/100 g FA), stearic acid (C18:0) (12.97–14.98 g/100 g FA) and linoleic acid (C18:2 n − 6) (10.31–11.93 g/100 g FA) (Table 4). These results agree with that reported by Barros et al. [4], who described the lipid profile of beef patties composed mainly of oleic acid (C18:1 n − 9; 36–48 g/100 g FA), palmitic acid (C16:0; 17–23 g/100 g FA) and stearic acid (C18:0; 12–14 g/100 g FA). The inclusion of maqui leaf powders produced a significant change in stearic, oleic, and linoleic fatty acids at the beginning and end of storage (*p* < 0.05). On contrary, the rest of the fatty acids showed the same or very similar values between beef patties from all treatments. For example, at the beginning of storage, stearic acid was found in higher concentration for the maqui powder treatments and lower in the control group on both study days. Despite these differences, the values were similar between samples, which produce low changes in the nutritional quality of reformulated beef patties. Moreover, this does not negatively affect the nutritional profile of the beef patties because stearic acid, despite being an SFA, has been shown to reduce Low Density Lipoproteins (LDL) levels in blood plasma and has no effect on High Density Lipoproteins HDL cholesterol [61,62]. Conversely, oleic and linoleic acid had lower proportions in treatments with natural antioxidants (*p* < 0.05). Once again, although the values are significantly different, the values were very similar among all treatments (variations were <1.9 and <0.8 g/100 g FA for C18:1 n – 9 and C18:2 n – 6, respectively). These differences could be related to the contribution of small amounts of stearic acid by the maqui leaf powder, which would affect the content of the other two main fatty acids (oleic and linoleic), present a dilution effect, and have a proportional lower content in the reformulated beef patties.

In addition, minor variations (not significant as discussed before; Table 1) in the fat content between the different batches, or variations in the proportions of fat/lean during the formulation and manufacture of the beef patties could be another source to explain these variations in FA of the beef patties. Even so, taking into account the enormous similarity between the data, it can be stated that the variations are minimal and without an apparent influence on the nutritional quality of the beef patties.

Logically, these minor differences were reflected in the total proportion of SFA, MUFA, and PUFA. The inclusion of maqui powders produced a significant increase in SFA concentration (*p* < 0.05) compared to the control group and synthetic antioxidant treatments. In contrast, a significant reduction of total MUFA was observed with the inclusion of maqui leaf powder. However, although some differences were observed in PUFA content, there is no clear trend, and the variations due to the maqui leaf powder are not clear. There is very limited information about the influence of the addition of leaf powder on the total fatty acids content of beef patties. In this sense, our findings agree with those reported by other authors, who also found no significant differences or observed minor changes in the fatty acids profile of sheep beef patties when pitanga leaf extract was added [21].

Regarding the nutritional indexes, the increase of n − 6 and/or the decrease of n − 3 FA in the samples with maqui powder was reflected in the n − 6/n − 3 ratio, showing a better proportion for the control and plus color beef patties However, it is important to highlight that this difference was not significant in beef patties on day 0, and only significant on Ma500 and Ma1000 at day 7, which confirms the lack of negative effect of the reformulation. The atherogenic (AI) and thrombogenic (TI) indices also increased proportionally with the increasing concentration of maqui leaf powder. These indices estimate the quality of the diet in nutritional terms. The most detrimental fatty acids related to AI and TI are lauric (C12:0), myristic (C14:0), and palmitic (C16:0), since they have a greater influence on LDL formation [63]. In particular, thrombogenicity is promoted by excessive consumption of polyunsaturated fatty acids of the n − 6 series because they have a greater facility for favoring thrombus development [64]. Ulbricht and Southgate [63] estimated that a diet can be considered healthy when AI < 1.0 and TI < 1.3. In this sense, our results indicate that AI and TI are within the established range. Therefore, the inclusion of maqui leaf powder does not negatively alter the fatty acid profile of the beef patties, although minor changes were detected in some individual FA content.

### 3.6. Sensory Analysis

**Figure 2:**
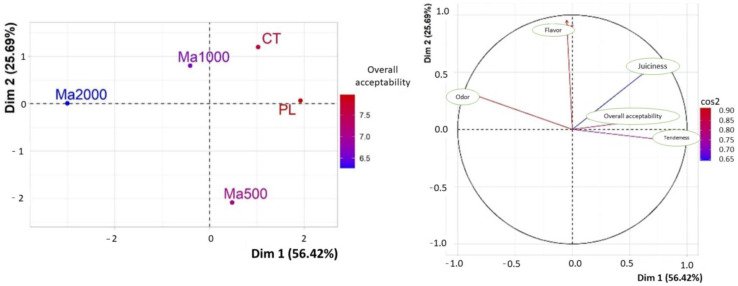
Principal Component Analysis (PCA), analysis of samples (CT, PL, Ma500, Ma1000, Ma2000), and analysis of variables (Odor, Flavor, Tenderness, Juiciness, and Overall Acceptability).

The sensory characteristics of beef patties were evaluated by 8 experienced members of the Meat Technology Center of the Universidad de La Frontera on day 0. Juiciness was the only parameter that differed significantly among treatments (*p* < 0.05) and was affected by the inclusion of 2000 ppm of maqui powder. The panelists assigned this treatment the lowest scores, which corresponded to less juiciness while the highest scores (more juiciness) were assigned to the Ma1000, Control, Plus color, and Ma500 treatments, in that order (Figure 2).

However, when a global comparison was drawn between all the parameters analyzed, it was found that in general terms, the inclusion of maqui leaf powder did not significantly affect the sensory attributes of the beef patties (*p* > 0.05) (Figure 2). A PCA of the adjusted means of the sensory evaluations of the samples was performed where the first two dimensions represent 82.11% of the inertia of the model. Strong discrimination of Ma2000 (0.952) was achieved with the variable Odor, opposing PL (0.759) and CT (0.316), which were more linked to Global Acceptability and Tenderness in Dimension 1 (56.42%). In Dimension 2 (25.69%), a strong negative correlation was evident for Ma500 (0.904) distancing it from the Flavor value, which was better correlated with CT (0.433) and Ma1000 (0.249). No significant differences were observed in tenderness, odor, flavor, and overall acceptability. Our results agree with those of other authors who have studied beef patties reformulation with moringa, walnut, and mesquite extracts or powders [45,64,65]. According to the evidence, it is possible to elaborate functional meat products by replacing synthetic antioxidants with maqui leaf powders without affecting their organoleptic characteristics.

## 4. Conclusions

This research demonstrates that maqui (*Aristotelia chilensis*) leaf powder did not modify the proximate composition of beef patties, while it has a strong inhibition effect against lipid oxidation. The fatty acids analysis showed significant differences in beef patties among treatments, although the results were very similar, and without any influence on the nutritional quality of beef patties, which were within the recommended values for a “healthy diet”. From the organoleptic point of view, the overall acceptability of the beef patties did not differ significantly among the treatments. In view of all the data obtained, the inclusion of maqui leaf powder improved the quality parameters and extended the shelf life of the patties. The application of 1000 ppm of this powder presented the best results among all other concentrations and could be the best option for the reformulation of meat patties. Therefore, maqui leaf powders could be a potential substitute for synthetic antioxidants for the production of functional, clean-label beef patties with an extended shelf life. However, due to the limited information on the antimicrobial potential of maqui leaves, future studies are recommended to evaluate the effect of maqui leaf polyphenols on the growth of pathogenic and spoilage microorganisms in meat products.

## Figures and Tables

**Table 1:** Total polyphenol content, main phenolic compounds, and antioxidant activity of *A. chilensis* leaf powder.

***Polyphenol content***	
Total phenolic content (TPC)* (mg GAE/g dm)	148.76 ± 19.03
Hydroxybenzoic acids (mg GAE/g) ^1^	82.5 ± 3.11
Hydroxycinnamic acids (mg CAE/g) ^2^	3.65 ± 0.07
Flavonoids (mg QE/g) ^3^	7.1 ± 0.04
***Antioxidant activity***	
**DPPH** **^•^** **(%)**	
325 µg/mL	94 ± 0.21
155 µg/mL	82 ± 0.57
10 µg/mL	15 ± 0.88
**IC_50_ DPPH (µg/mL)**	

The concentration of total polyphenols (TPC), main groups of phenolic compounds, and antioxidant activity (mean ± standard deviation) of 3 repetitions were assessed in three independent experiments; ^1^ Gallic acid equivalent (GAE); ^2^ Caffeoylquinic acid equivalent (CAE); ^3^ Quercetin equivalent (QE).

**Table 2:** Effect of antioxidant treatment on the proximate composition of beef patties.

Treatment	Moisture (%)	Fat (%)	Ash (%)
CT	63.22 ± 1.58	22.50 ± 0.71	2.23 ± 0.41
PL	61.10 ± 1.66	22.00 ± 0.50	2.30 ± 0.11
Ma500	64.25 ± 3.82	21.50 ± 0.71	1.91 ± 0.23
Ma1000	61.39 ± 1.76	22.50 ± 0.72	1.78 ± 0.10
Ma2000	63.16 ± 2.74	21.50 ± 0.70	2.10 ± 0.12

CT: control beef patties formulated without antioxidants; PL: beef patties formulated with synthetic antioxidant Plus color^®^; Ma500: beef patties formulated with 500 mg/kg of maqui leaf powder; Ma1000: beef patties formulated with 1000 mg/kg of maqui leaf powder; Ma2000: beef patties formulated with 2000 mg/kg of maqui leaf powder. No significant differences were observed for any parameter.

**Table 3:** Effect of antioxidant treatment and storage time on pH and color parameters of beef patties.

Parameter	Treatment	Storage Time	
		Day 0	Day 7
**pH**	CT	5.50 ± 0.08 ^a1^	5.65 ± 0.07 ^a2^
	PL	5.76 ± 0.01 ^b1^	5.27 ± 0.07 ^b2^
	Ma500	5.73 ± 0.01 ^b1^	5.33 ± 0.05 ^c2^
	Ma1000	5.62 ± 0.13 ^a1^	5.30 ± 0.03 ^d2^
	Ma2000	5.75 ± 0.07 ^b1^	5.39 ± 0.06 ^e2^
**Color**			
a*	CT	23.70 ± 1.28 ^a1^	7.62 ± 0.64 ^a2^
	PL	23.55 ± 2.65 ^a1^	8.33 ± 0.96 ^a2^
	Ma500	22.90 ± 2.02 ^a1^	6.78 ± 1.30 ^a2^
	Ma1000	19.15 ± 3.43 ^b1^	6.12 ± 0.82 ^b2^
	Ma2000	18.45 ± 1.46 ^c1^	4.77 ± 1.50 ^c2^
b*	CT	20.30 ± 1.23 ^a^	18.62 ± 2.42 ^a^
	PL	19.97 ± 2.31 ^a^	18.08 ± 2.42 ^a^
	Ma500	18.72 ± 1.08 ^a1^	13.88 ± 1.28 ^b2^
	Ma1000	19.35 ± 1.68 ^a1^	12.68 ± 1.45 ^b2^
	Ma2000	18.05 ± 2.36 ^a1^	11.15 ± 1.42 ^b2^
L*	CT	45.80 ± 2.17 ^a^	42.47 ± 2.84 ^a^
	PL	47.58 ± 4.82 ^a^	42.35 ± 2.05 ^a^
	Ma500	42.45 ± 1.78 ^a1^	34,72 ± 1.66 ^b2^
	Ma1000	46.07 ± 5.76 ^a1^	34.12 ± 2.61 ^b2^
	Ma2000	43.60 ± 2.96 ^a1^	34.83 ± 3.08 ^b2^

CT: control beef patties formulated without antioxidants; PL: beef patties formulated with synthetic antioxidant Plus color^®^; Ma500: beef patties formulated with 500 mg/kg of maqui leaf powder; Ma1000: beef patties formulated with 1000 mg/kg of maqui leaf powder; Ma2000: beef patties formulated with 2000 mg/kg of maqui leaf powder. ^a–e^ Different superscripts in the same column, and for the same parameter indicate significant differences (treatment effect; *p* < 0.05; Tukey’s test); ^1,2^ Different superscripts in the same raw (treatment), and for the same parameter indicate significant differences (storage time effect; *p* < 0.05; ANOVA).

**Table 4:** Effect of antioxidant treatment and storage time on fatty acids of beef patties.

Day 0						Day 7				
FA (%)	C	PL	Ma500	Ma1000	Ma2000	C	PL	Ma500	Ma1000	Ma2000
**C14:0**	1.55 ± 0.05 ^a1^	1.60 ± 0.00 ^bc1^	1.63 ± 0.00 ^c1^	1.64 ± 0.02 ^c1^	1.57 ± 0.00 ^ab1^	1.49 ± 0.00 ^a2^	1.53 ± 0.00 ^b2^	1.58 ± 0.00 ^c2^	1.62 ± 0.00 ^e1^	1.60 ± 0.00 ^d2^
**C14:1 n − 7**	0.16 ± 0.00 ^a1^	0.15 ± 0.00 ^a1^	0.15 ± 0.00 ^b1^	0.14 ± 0.00 ^b1^	0.12 ± 0.07 ^c1^	0.15 ± 0.00 ^a2^	0.14 ± 0.00 ^b2^	0.13 ± 0.00 ^b2^	0.14 ± 0.00 ^b1^	0.14 ± 0.00 ^b2^
**C16:0**	21.95 ± 0.04 ^a1^	22.26 ± 0.02 ^ab1^	22.81 ± 0.01 ^b1^	22.81 ± 0.33 ^b1^	22.59 ± 0.06 ^b1^	22.61 ± 0.05 ^a2^	22.79 ± 0.01 ^ab2^	22.97 ± 0.01 ^bc2^	23.11 ± 0.11 ^c1^	23.87 ± 0.09 ^d2^
**C16:1 n − 7**	3.15 ± 0.00 ^a1^	3.18 ± 0.04 ^a1^	2.91 ± 0.00 ^b1^	3.11 ± 0.04 ^a1^	2.77 ± 0.02 ^c1^	2.98 ± 0.01 ^a2^	2.98 ± 0.01 ^a2^	2.83 ± 0.00 ^b2^	2.98 ± 0.03 ^a1^	2.68 ± 0.00 ^c2^
**C17:0**	0.48 ± 0.00 ^a1^	0.47 ± 0.04 ^a1^	0.42 ± 0.00 ^ab1^	0.46 ± 0.00 ^ab1^	0.38 ±0.03 ^b1^	0.48 ± 0.00 ^a2^	0.41 ± 0.00 ^b1^	0.41 ± 0.00 ^b2^	0.44 ± 0.03 ^ab1^	0.44 ± 0.01 ^ab1^
**C17:1 n − 7**	0.45 ± 0.00 ^a1^	0.45 ± 0.00 ^a1^	0.35 ± 0.00 ^b1^	0.36 ± 0.00 ^b1^	0.31 ± 0.00 ^c1^	0.43 ± 0.00 ^a2^	0.41 ± 0.00 ^b2^	0.34 ± 0.00 ^c2^	0.34 ± 0.00 ^c2^	0.35 ± 0.00 ^c2^
**C18:0**	12.97 ± 0.06 ^a1^	13.15 ± 0.02 ^a1^	14.27 ± 0.02 ^c1^	13.80 ± 0.19 ^b1^	14.14 ± 0.01 ^bc1^	13.24 ± 0.01 ^a2^	13.21 ± 0.01 ^a1^	14.18 ± 0.00 ^b2^	14.19 ± 0.03 ^b1^	14.98 ^c^ ± 0.05 ^c2^
**C18:1 n − 9**	44.07 ± 0.21 ^ab1^	44.24 ± 0.01 ^a1^	43.15 ± 0.00 ^bc1^	42.67 ± 0.52 ^c1^	43.48 ± 0.01 ^abc1^	44.03 ± 0.05 ^a1^	44.10 ± 0.02 ^a2^	42.88 ± 0.03 ^b2^	42.37 ± 0.16 ^c1^	42.65 ± 0.19 ^bc2^
**9 t-C18:1**	0.20 ± 0.00 ^a1^	0.21 ± 0.00 ^b1^	0.16 ± 0.00 ^c1^	0.17 ± 0.00 ^d1^	0.15 ± 0.07 ^e1^	0.19 ± 0.00 ^a2^	0.20 ± 0.00 ^a2^	0.16 ± 0.00 ^b2^	0.15 ± 0.01 ^b1^	0.17 ± 0.01 ^ab1^
**C18:2 n − 6**	11.93 ± 0.25 ^a1^	11.32 ± 0.00 ^b1^	11.22 ± 0.02 ^b1^	11.88 ± 0.11 ^a1^	11.68 ± 0.04 ^ab1^	11.54 ± 0.00 ^bc1^	11.40 ± 0.05 ^b1^	11.71 ± 0.05 ^cd2^	11.77 ± 0.08 ^d1^	10.31 ± 0.03 ^a2^
**C18:3 n − 6**	0.21 ± 0.00 ^a1^	0.20 ± 0.00 ^ab1^	0.20 ± 0.00 ^bc1^	0.19 ± 0.00 ^c1^	0.21 ± 0.00 ^a1^	0.21 ± 0.00 ^a2^	0.21 ± 0.00 ^b2^	0.20 ± 0.00 ^c2^	0.20 ± 0.00 ^c1^	0.22 ± 0.00 ^a2^
**C20:0**	0.67 ± 0.00 ^a1^	0.59 ± 0.01 ^b1^	0.59 ± 0.02 ^b1^	0.64 ± 0.01 ^ab1^	0.61 ± 0.00 ^b1^	0.62 ± 0.00 ^a2^	0.58 ± 0.00 ^c1^	0.60 ± 0.00 ^b1^	0.61 ± 0.00 ^a1^	0.54 ± 0.00 ^d2^
**C18:3 n − 3**	0.71 ± 0.05 ^a1^	0.69 ± 0.05 ^a1^	0.60 ± 0.00 ^a1^	0.59 ± 0.01 ^a1^	0.64 ± 0.00 ^a1^	0.66 ± 0.00 ^a1^	0.65 ± 0.00 ^b1^	0.61 ± 0.00 ^c2^	0.57 ± 0.00 ^d1^	0.58 ± 0.00 ^d2^
**CLA**	0.13 ± 0.01 ^a1^	0.11 ± 0.01 ^ab1^	0.12 ± 0.00 ^ab1^	0.12 ± 0.00 ^ab1^	0.11 ± 0.00 ^b1^	0.12 ± 0.00 ^b1^	0.11 ± 0.00 ^a1^	0.11 ± 0.00 ^a2^	0.13 ± 0.00 ^c2^	0.13 ± 0.00 ^c2^
**C21:0**	0.52 ± 0.00 ^a1^	0.50 ± 0.00 ^b1^	0.48 ± 0.00 ^c1^	0.49 ± 0.00 ^bc1^	0.50 ± 0.00 ^b1^	0.51 ± 0.00 ^a1^	0.50 ± 0.00 ^a1^	0.50 ± 0.00 ^a2^	0.49 ± 0.00 ^a1^	0.50 ± 0.00 ^a1^
**C20:2 n − 6**	0.11 ± 0.00 ^a1^	0.11 ± 0.00 ^a1^	0.12 ± 0.00 ^b1^	0.12 ± 0.00 ^b1^	0.11 ± 0.00 ^a1^	0.10 ± 0.00 ^a2^	0.11 ± 0.00 ^b2^	0.11 ± 0.00 ^c2^	0.12 ± 0.00 ^c2^	0.11 ± 0.00 ^bc1^
**C22:0**	0.35 ± 0.06 ^ab1^	0.38 ± 0.00 ^ab1^	0.40 ± 0.00 ^b1^	0.41 ± 0.01 ^b1^	0.29 ± 0.00 ^a1^	0.28 ± 0.01 ^a1^	0.33 ± 0.05 ^ab1^	0.29 ± 0.00 ^ab2^	0.38 ± 0.00 ^b2^	0.36 ± 0.00 ^ab2^
**C20:5 n − 3**	0.10 ± 0.00 ^a1^	0.10 ± 0.00 ^a1^	0.10 ± 0.00 ^a1^	0.10 ± 0.00 ^a1^	0.09 ± 0.00 ^b1^	0.09 ± 0.00 ^ab2^	0.10 ± 0.00 ^bc1^	0.10 ± 0.00 ^bc2^	0.10 ± 0.00 ^c1^	0.10 ± 0.00 ^a1^
**C24:1 n − 9**	0.13 ± 0.01 ^a1^	0.12 ± 0.01 ^a1^	0.13 ± 0.00 ^a1^	0.13 ± 0.01 ^a1^	0.12 ± 0.00 ^a1^	0.13 ± 0.00 ^b1^	0.11 ± 0.00 ^a1^	0.11 ± 0.00 ^a2^	0.12 ± 0.00 ^ab2^	0.11 ± 0.00 ^a1^
**SFA**	38.55 ± 0.02 ^a1^	39.01 ± 0.02 ^a1^	40.67 ± 0.01 ^b1^	40.30 ± 0.57 ^b1^	40.13 ± 0.04 ^b1^	39.27 ± 0.05 ^a2^	39.40 ± 0.05 ^a2^	40.60 ± 0.01 ^b2^	40.90 ± 0.11 ^b1^	42.36 ± 0.13 ^c2^
**MUFA**	48.16 ± 0.21 ^a1^	48.35 ± 0.04 ^a1^	46.86 ± 0.01 ^b1^	46.58 ± 0.48 ^b1^	46.94 ± 0.01 ^b1^	47.90 ± 0.04 ^a1^	47.94 ± 0.01 ^a2^	46.46 ± 0.03 ^b2^	46.10 ± 0.20 ^b1^	46.10 ± 0.18 ^b2^
**PUFA**	13.30 ± 0.19 ^a1^	12.63 ± 0.06 ^bc1^	12.47 ± 0.02 ^c^	13.13 ± 0.09 ^a1^	12.93 ± 0.03 ^ab1^	12.83 ± 0.01 ^bc1^	12.66 ± 0.05 ^b1^	12.94 ± 0.04 ^c2^	13.01 ± 0.09 ^c1^	11.55 ± 0.04 ^a2^
**n − 3**	0.84 ± 0.06 ^a1^	0.82 ± 0.05 ^a1^	0.74 ± 0.00 ^a1^	0.73 ± 0.01 ^a1^	0.76 ± 0.01 ^a1^	0.79 ± 0.00 ^a1^	0.78 ± 0.00 ^a1^	0.75 ± 0.00 ^b2^	0.71 ± 0.01 ^c1^	0.71 ± 0.00 ^c2^
**n − 6**	12.32 ± 0.25 ^a1^	11.70 ± 0.01 ^b1^	11.61 ± 0.02 ^b1^	12.28 ± 0.10 ^a1^	12.06 ± 0.04 ^ab1^	11.92 ± 0.01 ^bc1^	11.77 ± 0.05 ^b1^	12.09 ± 0.04 ^cd2^	12.16 ± 0.08 ^d1^	10.71 ± 0.04 ^a2^
**n − 6/n − 3**	14.67 ± 1.25 ^a1^	14.30 ± 0.93 ^a1^	15.63 ± 0.01 ^a1^	16.79 ± 0.41 ^a1^	15.79 ± 0.17 ^a1^	15.14 ± 0.01 ^a1^	15.11 ± 0.09 ^a1^	16.20 ± 0.07 ^b2^	17.10 ± 0.01 ^c1^	15.19 ± 0.04 ^a2^
**AI**	0.46 ± 0.00 ^a^	0.47 ± 0.00 ^ab^	0.50 ± 0.00 ^c^	0.49 ± 0.01 ^c^	0.48 ± 0.00 ^bc^	0.47 ± 0.00 ^a2^	0.48 ± 0.00 ^a2^	0.50 ± 0.00 ^b2^	0.50 ± 0.00 ^b1^	0.53 ± 0.00 ^c2^
**TI**	1.11 ± 0.01 ^a^	1.14 ± 0.00 ^a^	1.23 ± 0.00 ^b^	1.21 ± 0.03 ^b^	1.20 ± 0.00 ^b^	1.15 ± 0.00 ^a2^	1.16 ± 0.00 ^a2^	1.23 ± 0.00 ^b1^	1.24 ± 0.01 ^b1^	1.32 ± 0.01 ^c2^

SFA: saturated fatty acids; MUFA: monounsaturated fatty acids; PUFA: polyunsaturated fatty acids. AI (atherogenic index): C12:0 + (4 × C14:0) + C16:0/((Σ n − 3 + Σ n − 6) + Σ MUFA). TI (thrombogenic index): (C14:0 + C16:0 + C18:0)/((0.5 × Σ MUFA) + (0.5 × Σ n − 6) + (0.5 × Σ n − 3) + (Σ n − 3/Σ n − 6)). Results are shown as the mean ± standard deviation. ^a–e^ Different superscripts in the same day, and for the same fatty acid indicate significant differences (treatment effect; *p* < 0.05; Tukey’s test); ^1,2^ Different superscripts in the same treatment, and for the same fatty acid indicate significant differences (storage time effect; *p* < 0.05; AVONA).

## Data Availability

Data are contained within the article.
